# *Grevillea robusta* Delayed the Progression of Experimentally Induced Hepatic Fibrosis and Cirrhosis in Wistar Rats by Attenuating the Expression of Smooth Muscle Actin, Collagen, and TGF-β

**DOI:** 10.3389/fphar.2022.904584

**Published:** 2022-06-15

**Authors:** Saaid Hameed, Atta Ur Rehman, Shazma Massey, Nawazish-i-Husain Syed, Fareeha Anwar, Dildar Ahmed, Sarfraz Ahmad

**Affiliations:** ^1^ Faculty of Pharmacy, The University of Lahore, Lahore, Pakistan; ^2^ Department of Pharmacy, Faculty of Natural Sciences, Forman Christian College, Lahore, Pakistan; ^3^ Department of Chemistry, Faculty of Natural Sciences, Forman Christian College, Lahore, Pakistan; ^4^ Punjab University College of Pharmacy, University of the Punjab, Lahore, Pakistan; ^5^ Riphah Institute of Pharmaceutical Sciences, Lahore Campus, Lahore, Pakistan; ^6^ Department of Chemistry, Faculty of Science, University of Malaya, Kuala Lumpur, Malaysia; ^7^ Department of Chemistry and Biochemistry, University of Windsor, Windsor, ON, Canada

**Keywords:** *Grevillea robusta*, fibrosis, alpha-SMA, collagen, oxidative stress

## Abstract

The chronic damage to the liver causes fibrosis, especially when different proteins are accumulated in the liver, which is the basic characteristic of chronic liver damage. The excessive accumulation of the matrix protein such as collagen causes liver fibrosis. Liver fibrosis leads to cirrhosis, liver failure, and portal vein hypertension. Plants having antioxidants, free radical scavenging activities, and anti-inflammatory constituents are believed to be hepatoprotective in nature. *Grevillea robusta* (GR) is native to the subtropical environment. Its *in vitro* antioxidant, cytotoxic, and free radical scavenging activities are known, while the effect on liver fibrosis and cirrhosis remains elusive. The aim of this study was to evaluate the hepatoprotective and antifibrotic effects of *Grevillea robusta* plant. GR leaf extract (GREE) was prepared from the hydroethanolic extract (70%). Polyphenol and flavonoid contents and the *in vitro* antioxidant activity of the extract were determined. *In vivo* hepatitis was induced in Wistar rats by continual IP injections of CCl_4_. GREE was administered by oral gavage at a dose of 100, 300, and 500 mg/kg of body weight once daily for 4 weeks. Variations in rat’s body weight, liver-to-body weight ratio, serum alanine aminotransferases, gamma-glutamyltransferase, liver histology, and cellular markers of liver fibrosis were evaluated. Serum levels of alanine aminotransferase (ALT) (*p < 0.05*) and gamma-glutamyltransferase (γ-GT) (*p < 0.001*) were decreased in the treatment group compared with the disease control group. RBC count was increased (*p* < 0.001) in the treatment group compared with the disease control group. The expression of alpha-SMA was downregulated to 40% (*p* < 0.05) and that of collagen was decreased by 9% (*p* < 0.05) compared with the disease control group. Extracellular matrix deposition and necrotic areas were also decreased as compared to the disease control group. It can be concluded that GR possesses hepatoprotective action by virtue of antioxidant constituents and delays the progression of liver cirrhosis by suppressing the activation of extracellular matrix–producing cells in the liver.

## Introduction

The liver is a vital organ that carries out important functions in the human body. Liver cells constantly come across many chemicals that have oxidative potential. Oxidative stress plays an inevitable role during the inflammatory process of hepatitis. Oxidative stress on a small scale is manageable; however, prolonged oxidative stress in the liver tends to change the structure and function of the parenchymal tissue; consequently, it leads to liver fibrosis and cirrhosis (Bataller and Brenner, 2005; Rehman, 2014). Repeated exposure to toxins and bouts of hepatic necrosis and inflammation are the underlying causes of liver fibrosis (Ballestri et al., 2021).

On a molecular level, fibrosis is characterized by the hepatic stellate cell (HSC) activation, a major source of fibrotic scar generation (Kisseleva and Brenner, 2021). In a healthy liver, HSC is found in a quiescent form, which becomes activated due to drugs, microbial infections, and metabolic abnormalities (Winkler et al., 2021). Oxidative stress plays an important role in the process of HSC activation (Dunning et al., 2013). The HSC proliferation rate is enhanced, and the induction of alpha-smooth muscle actin (α-SMA) and collagen (Coll) is evident. The activation of HSC is regulated by diverse types of inflammatory cytokines, which are produced by liver-resident macrophages, hepatocytes, and endothelial cells and also by HSC themselves (Friedman, 2003; Canbay et al., 2004; Cai et al., 2020). These cytokines include tumor necrosis factor-alpha (TNF-α) and growth factors, such as platelet-derived growth factors and transforming growth factor-beta (TGF-β) (Tiggelman et al., 1995). This is why α-SMA and Coll are considered one of the molecular markers of HSC activation, and therefore, HSC is considered as a therapeutic target for liver fibrosis and cirrhosis (Wu and Zern, 2000; Bataller and Brenner, 2001).

Natural drugs have been used by folks in the subcontinent region. It is due to their cost-effectiveness and less untoward effects. Several studies have proved that the polyphenols and flavonoids in the extracts of the plant exhibited antioxidant and anti-inflammatory properties and thus imparted hepatoprotection against chemically induced hepatitis (Gebhardt, 2002; Luk et al., 2007; Rehman et al., 2017). *Grevillea robusta* (*G. robusta*), commonly known as silky oak, belongs to the family Proteaceae and is commonly found in subtropical and dry rainforest geographical areas of the world. A variety of active principles have been isolated from the plant and reported to show antioxidant, antiproliferative, and tyrosinase inhibitory activities (Cannon et al., 1970; Cannon et al., 1973; Chuang and Wu, 2007; Chuang et al., 2011). However, its pharmacological activities as a hepatoprotective and an antifibrotic agent have never been explored. Therefore, this study aimed to screen phytochemicals present in the crude extracts of the leaves of *G. robusta* and their potential to prevent liver fibrosis and cirrhosis in an animal model.

## Materials and Methods

### Plant Collection

The leaves of *G. robusta* were collected from the botanical garden of the Government College University (GCU), Lahore, Pakistan. The specimen was identified, and the voucher number GC.Herb.Bot.2915 was deposited at the herbarium of the university.

### Preparation of the Ethanolic Extract of *Grevillea robusta* (GREE)

**FIGURE 1:**
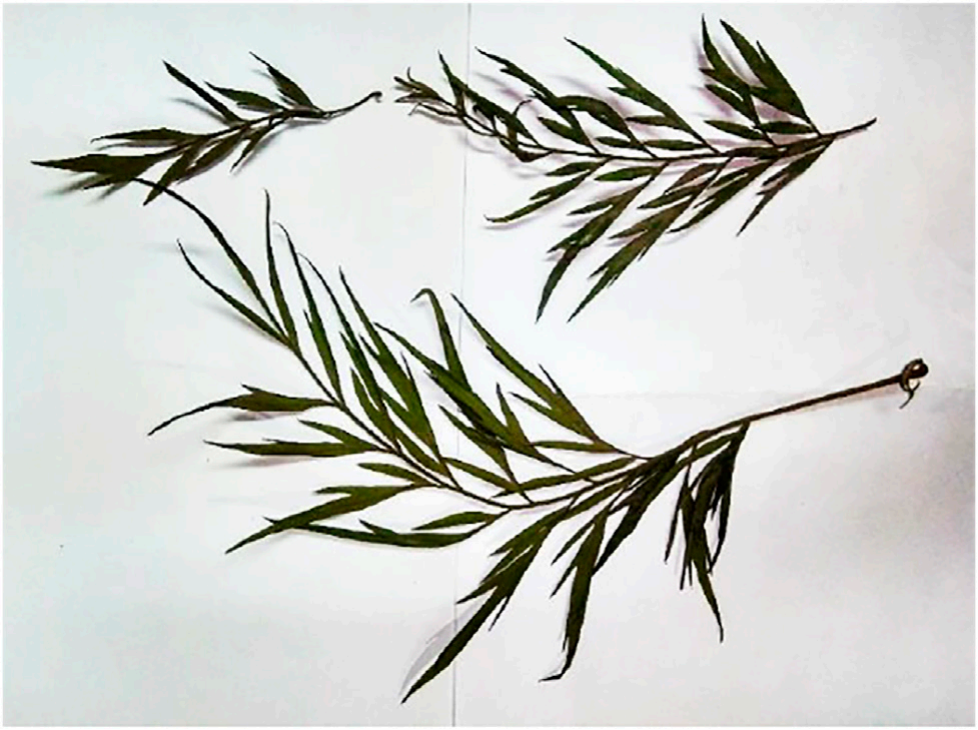
Semi-dried leaves of *G. robusta*.

The crude extract was prepared by the maceration method. Briefly, fresh leaves (Figure 1) were rinsed with tap water and shade-dried for 5 days. The dried leaves were powdered in a grinder. A known amount of the powder (800 g) was macerated in a closed container using a 2.5 L mixture of ethanol and water (70:30). After 7 days, the extract was recovered by filtration with a double-layered muslin cloth followed by vacuum filtration through a Whatman filter paper No. 40. The extract was then concentrated to semisolid consistency using a rotary evaporator (Heidolph, Germany) under vacuum at 30°C. Afterward, the extract was completely dried at room temperature and then stored in amber-colored vials in a refrigerator (2-8°C).

### Phytochemical Analysis

#### Determination of the Total Phenolic Content

The total phenolic content of the hydroethanolic extract of *G. robusta* leaves (GREE) was determined by an already-established protocol (Ahmed et al., 2014). Briefly, a stock solution (1 mg/ml) of GREE was prepared. A measure of 40 µl of the GREE stock solution was mixed with 3.16 ml distilled water with the subsequent addition of 200 µl Folin–Ciocalteu reagent (FCR). This mixture was placed in an incubator for 8 min in the dark. After incubation, 600 µl of sodium carbonate solution (7%) was added and mixed thoroughly, followed by incubation for 30 min at 40°C. The absorbance at 765 nm was measured against blank. The calibration curve of gallic acid was drawn, and TPC was expressed as µg/ml of the gallic acid equivalent (Aryal et al., 2019).

#### Determination of the Total Flavonoid Content

The total flavonoid content of GREE was quantified by a standard method (Ahmed et al., 2014). Briefly, 300 µl of stock solution was mixed with 3.4 ml of 30% methanol, followed by the addition of 150 µl of sodium nitrite (0.5 M) and 150 µl of aluminum chloride (0.3 M) solution. After 5 min of incubation, 1 ml of sodium hydroxide (1.0 M) was added and mixed well (Dirar et al., 2019). The absorbance was measured at 506 nm alongside a blank. Rutin quantification was taken as a standard. The flavonoid content was evaluated as µg/ml of the rutin equivalent (RE).

#### GCMS Analysis

The GCMS of the GREE was carried out using Agilent Technologies 7890A GC System and 5975C inert MSD Detector, HP-5MS column, using 30 m × 0.25 mm × 0.25 µm fused capillary silica tubing. Mass spectra and chromatograms were analyzed using the NIST 5 software. The temperature protocol for the GCMS detection was as follows: injection port temperature was 200°C, and the helium flow rate was 1 ml/min. The oven temperature was programmed from 60°C with an increment of 10°C/min to 310°C, and this temperature was maintained for 5 min. The ionization voltage was set at 70ev. The sample was injected in splitless mode, and the spectral scan range was set at 45–500 (MHz). The total GC running time was 30 min (Sermakkani and Thangapandian, 2012). The fragmentation patterns of the mass spectra were compared (head to tail) with those of the known compounds stored in the NIST library.

#### Determination of the DPPH Radical Scavenging Activity

The antioxidant activity of GREE was estimated by the DPPH radical scavenging assay (Trinh et al., 2020). In this method, 3 ml of DPPH solution (having an optical density of 0.98 at 517 nm) was mixed with 100 µl GREE stock solution or the standard solution (BHT). The solution was then incubated at 37°C for 30 min, followed by the measurement of absorbance at 517 nm. The antioxidant activity of the extract was calculated using the formula:

% Activity=[(Ac-As)/Ac]×100,

where A_c_ is the absorbance of the control (it is the absorbance of the reaction mixture without the sample or standard), and A_s_ is the absorbance of the sample. EC_50_ was measured using the regression curve Y = 0.8257x-2.2579.

### *In Vivo* Hepatoprotective Activity

The *in vivo* experimental procedure was approved by the Research Ethics Committee, the University of Lahore, Lahore, Pakistan, with void letter no. IAEC 2014–003. Wistar rats of either sex (*n* = 16, weighing 150–200 g) were first acclimatized to standard laboratory environmental conditions. They were fed with commercial chow and water ad libitum. They were randomly selected into groups as follows: group A (control group), in which animals were administered 1 µl corn oil/gram of body weight (gbw) by intraperitoneal (IP) injection (twice a week for 6 weeks); group B (disease group), in which liver fibrosis was induced by IP injection of CCl_4_ twice weekly, according to the established protocol described before (14). Briefly, CCl_4_ was dissolved in corn oil (1:10) and injected at a dose of 0.5 and 0.75 µl CCl_4_/gbw for the first and second week, respectively, while from the third week until the sixth week, 1 µl CCl_4_/gbw was administered; group C, in which animals were administered GREE orally by using a gastric gavage tube (i.g) from third to sixth week daily; and treatment groups, groups D, E, and F, in which animals were treated with an extract at a dosage of 100, 300, and 500 mg/kgbw, respectively; in other words, starting from the third week, animals were treated with GREE at the indicated doses, while the administration of CCl_4_ is continued similar to the disease group. After 24 h of the last dose, animals were anatomized under chloroform anesthesia. Blood samples were withdrawn by cardiac puncture for the measurement of complete blood count and other biochemical parameters. In addition, livers were also excised and washed in normal saline and weighed. Afterward, 1-cm^3^ portion of the liver tissue was removed from the largest lobe of liver and preserved in 10% formalin for histopathological analysis. The remaining part from the same lobe was stored at −80°C for RNA isolation.

### Quantification of Biochemical Parameters

The quantification of serum ALT, AST, and GGT was determined by using diagnostic kits (LABTEST, France), by following the manufacturer’s protocols as described previously (Atta et al.).

### Measurement of Blood Cell Indices

The anticoagulant treated blood was analyzed for blood cell indices on an automated cell analyzer (Roche, Switzerland), according to the manufacturer’s protocol.

### Histopathological Evaluation

The liver tissue preserved in 10% formalin was then fixed in paraffin blocks; 5-µm-thick sections were cut and fixed on glass slides. Hematoxylin and eosin staining was performed according to the method described earlier (Atta et al.).

### Real-Time PCR Analysis

A quantitative gene expression analysis of alpha-smooth muscle actin (α-SMA) and collagen (Coll) was carried out following the protocol, as described before (Atta et al.).

### Statistical Analysis

All data are expressed as the mean ± standard deviation (SD). Statistically significant differences between the groups were analyzed by one-way analysis of variance (ANOVA), followed by Bonferroni’s descriptive test using the GraphPad Prism, version 5. P˂0.05 was considered statistically significant.

## Results

### Phytochemical Screening

**FIGURE 2:**
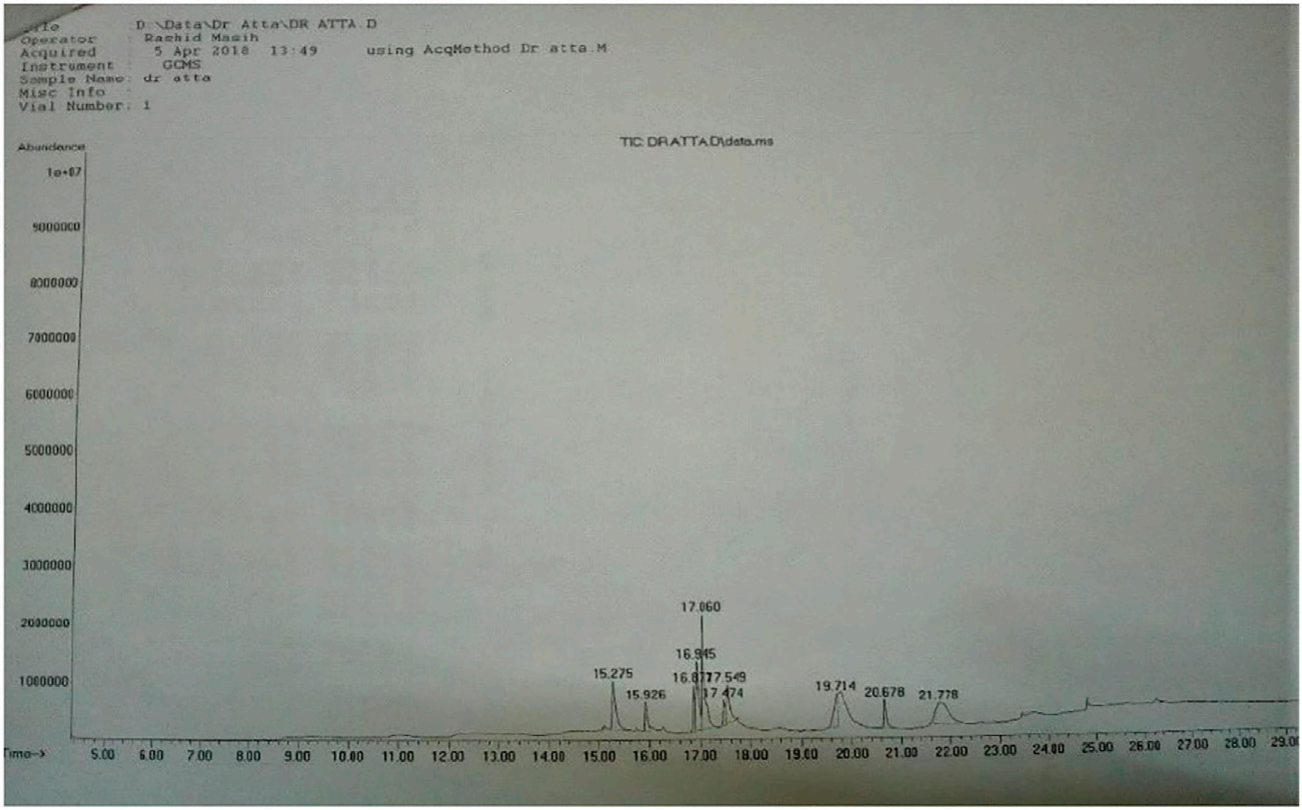
GCMS chromatogram of GREE.

Phytochemical screening of GREE showed the presence of plant secondary metabolites, phenolic, flavonoid, esters, and organic acids. GREE contained 270 ± 20.50 µg of gallic acid equivalent (GAE) per mg of dried mass and 153 ± 24.87 µg of RE per mg of dried mass (Table 1). However, the GCMS analysis confirmed the presence of esters and organic carboxylic acids (Figure 2 and Table 2).

**TABLE 1:** Total phenolic and flavonoid contents of GREE.

Phenolic content µg of the gallic acid equivalent/mg of the dried mass	Flavonoid content µg of the rutin equivalent/mg of the dried mass
270 ± 20.50	153 ± 24.87

The values are expressed as mean ± SD (*n* = 3)

**TABLE 2:** GCMS chromatograph of GREE.

Peak No.	Name	Mol Wt	Retention time	Corrected area	% of total
1	Hexadecanoic acid, methyl ester, C_17_H_34_O_2_	270	15.275	3936124	11.480
2	Hexadecanoic acid, ethyl ester, C_18_H_36_O_2_	284	15.926	1626122	4.743
3	9,12-Octadecadienoic acid, methyl ester, (E,E)-C_19_H_34_O_2_	294	16.877	1736998	5.066
4	(Z,Z,Z)-9,12,15-Octadecatrienoic acid, methyl ester, C_19_H_32_O_2_	292	16.945	414034	12.174
5	Phytol C_20_H_40_O	296	17.060	8082629	23.573
6	Linoleic acid ethyl ester, C_20_H_36_O_2_	308	17.474	988604	2.883
7	(Z,Z,Z)- 9,12,15-Octadecatrienoic acid, ethyl ester, C_19_H_32_O_2_	306	17.549	2801807	8.172
8	4,7,10-trimethyl-2,5,8,11-tetraoxatetradecan-13-ol C_13_H_28_O_5_	264	19.714	2913407	8.497
9	1,2-Benzenedicarboxylic acid, diisooctyl ester, C_24_H_38_O_4_		20.678	1577021	4.599
10	2-(3-Acetooxy-4,4,14-trimethylandrost-8-en-17-yl)-propionic acid, C_27_H_42_O_4_	430	21.778	6450349	18.813

The extract was dissolved in ethanol and filtered. Chromatogram and fragment analyses were conducted using the GCMS library, NIST-5.

### *In vitro* Antioxidant Activity

Table 3 shows that GREE possessed significant free radical scavenging activity compared with the standard antioxidant BHT (butylated hydroxyl toluene). BHT maximally scavenged 89.35% DPPH at 100 μg/ml concentration, while GREE maximally scavenged 68% DPPH at 100 μg/ml concentration. An effective concentration to scavenge 50% of the DPPH (EC_50_) of GREE was 74.26 ± 0.46 μg/ml, while EC_50_ of BHT was 41.23 ± 0.27 μg/ml (Table 3).

**TABLE 3:** DPPH scavenging activity of GREE and BHT

Concentration of BHT or GREE (ug/ml)	% scavenging activity	
	BHT	GREE
0	0.00	0.00
15	34.06 ± 0.84	6.03 ± 0.34
30	45.96 ± 0.58	15.49 ± 1.20
45	63.69 ± 3.24	27.93 ± 0.21
60	73.62 ± 0.63	40.91 ± 0.74
75	77.89 ± 0.32	51.35 ± 1.14
90	83.96 ± 0.21	61.93 ± 2.09
100	89.35 ± 0.33	68.10 ± 0.39
EC_50_	41.23 ± 0.27	74.26 ± 0.46

A dose-dependent effect of DPPH scavenging (%) of GREE (µg equivalent of the phenolic content) and comparison with BHT as the standard. EC_50_ is the effective dose, which scavenges 50% of the DPPH radicals. The highlighted gray ones are the values close to the EC_50_ values. The results are represented as mean ± SD of the assay in triplicate.

### *In Vivo* Test Results

#### Effect of GREE and CCl_4_ on Animal Weights

Table 4 shows rat’s body weight variations in response to different treatments over the course of the experiment. The average weight of animals in the control group increased by 22.5% by the end of the sixth week. Extract administration at a dose of 500 mg/Kgbw did not affect the health of rats; in this group, average body weight increased by 5.8%. In the CCl_4_ treatment group, the body weight was decreased by 8.9% compared with that at day 1. The treatment of the CCl_4_-challenged group with GREE at a dose of 100, 300, and 500 mg/kgbw caused an average increase in the body weights by 5.6%, 10.2%, and 8.8%, respectively.

**TABLE 4:** Effect of GREE on the body weight of animals.

Week	Control	GREE 500	CCl_4_	CCl_4_ +GREE 100	CCl_4_+GREE 300	CCl_4_+GREE 500
Week 1	192 ± 28.6 (0)	198.6 ± 15.5 (0)	185.2 ± 18.3 (0)	198.1 ± 10.3 (0)	200.2 ± 12.1 (0)	175.2 ± 9.1 (0)
Week 2	208 ± 36.9 (+8.3)	195 ± 8.6 (−1.8)	177 ± 19.6 (−4.3)	200.5 ± 1.1 (+1.2)	207.2 ± 8.1 (+3.4)	182.1 ± 8.6 (+3.9)
Week 3	222.2 ± 41.1(+15.6)	200 ± 21.7 (0.67)	181 ± 12.3 (−2.2)	202.2 ± 9.2 (+2.0)	213.2 ± 11.2 (+6.4)	187.2 ± 9.9 (+6.8)
Week 4	216 ± 41.6 (+12.5)	195.3 ± 12.7 (−1.6)	173.6 ± 14.0 (−4.5)	206 ± 11.9 (+3.9)	206 ± 10.9 (+2.8)	197 ± 12.3 (+12.4
Week 5	223.7 ± 48.9 (+16.5)	205.6 ± 14.5 (+3.5)	170.6 ± 12.9 (−6.2)	211.5 ± 10.2 (+5.6)	211.7 ± 9.8 (+10.2)	190.7 ± 10.8 (+8.8)
Week 6	235.2 ± 52.7 (+22.5)	210.3 ± 18.7 (+5.8)	168.7 ± 16.8 (−8.9)	220.7 ± 12.9 (+)	210.7 ± 10.8 (+10.2)	192.7 ± 11.3 (+9.9)

Each value is represented as mean ± S.D. Significance: *p < 0.001* when compared to group A and ^###^ *p < 0.001* when compared to group B.

#### Effect of GREE and CCl_4_ on the Serum Levels of Liver Damage Markers

**FIGURE 3:**
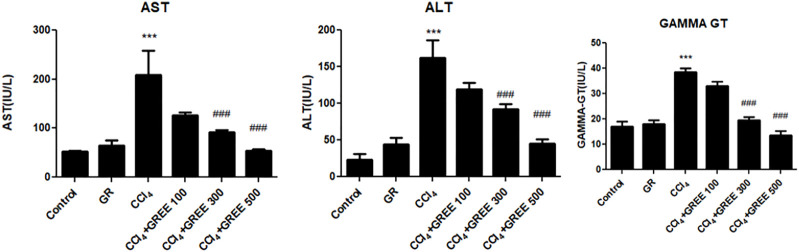
Quantification of liver damage markers in various treatment groups. Quantification of AST, ALT, and gamma-GT in the serum of animals from various treatment groups was determined by commercially provided diagnostic kits. Values are represented as the mean ± SD, *n* = 4. Significance: ***p < 0.001 when compared to group A and ^###^ *p < 0.001* compared to group B. Effect of GREE and CCl_4_ on blood indices.

Figure 3 shows the levels of serum biomarkers of liver damage (AST, ALT, and GGT) in rats in response to GREE and CCl_4_ administration at the 6th week of the experiment. In the control group, the levels of AST, ALT, and GGT remained lowest compared with those in all treatment groups. The administration of GREE at 500 mg/kg body weight did not elevate the levels compared with those in control groups. On the contrary, the CCl_4_ administration caused a significant (*p* < 0.001) increase in the serum levels of AST, ALT, and GGT (by fourfold, sixfold, and 2.5-fold, respectively) compared with those in the control group. Moreover, in rats of treatment groups, doses of 300 and 500 (mg/kg body weight) significantly reduced the serum levels of AST (50% and 75%, respectively), ALT (50% and 60%, respectively), and GGT (47% and 58%, respectively) as opposed to those in the CCl_4_ treatment group.

Table 5 shows the effect of various treatments on the levels of blood cell indices in response to GREE and CCl_4_. Data show that the CCl_4_ challenge led to an increase in white blood cell (WBC) count by twofold, while a significant decrease was observed in red blood cell count and hemoglobin levels. There was no effect of CCl_4_ on the hematocrit (HCT) and platelets’ count. In the control group, the mean WBC count was 14.3 × 10³/µl, which was elevated significantly (*p < 0.05*) to 31.2 × 10³/µl after the challenge with CCl_4_. In the control group, the RBC count was 7.6 × 10⁶/µl, whereas in the CCl_4_-treated group, the RBC count decreased to 5.7 × 10⁶/µl (*p < 0.001*). Furthermore, treatment with GREE at various doses in CCl_4_-intoxicated rats restored the count of RBC to 8.3 × 10⁶/µl (*p < 0.001*), while the WBC count was reduced to a minor extent.

**TABLE 5:** Hematological analysis of the treated groups.

Hematological parameter (unit)	Control	GREE 500	CCl_4_	CCl_4_ +GREE 100	CCl_4_+GREE 300	CCl_4_+GREE 500
WBC(×10³/µl)	14.3 ± 4.1	15.7 ± 1.1	31.2 ± 5.4*	28.9 ± 2.4	25.5 ± 10.8	20.3 ± 4.3
RBC (×10⁶/µl)	7.6 ± 0.4	8.0 ± 0.2	5.7 ± 0.7***	6.2 ± 0.51	8.3 ± 0.4###	8.7 ± 0.41
Hb (g/dl)	13.2 ± 0.5	14.1 ± 0.4	11.5 ± 0.4*	10.37 ± 0.28	13.5 ± 0.9##	13.6 ± 0.41
HCT (%)	39.7 ± 6.0	44.9 ± 0.4	43.6 ± 4.0	42.8 ± 2.4	44.8 ± 3.1	42.6 ± 2.7
PLT (×10³/µl)	895 ± 167.1	1060 ± 57.9	751.5 ± 51.1	813.1 ± 25.1	897.5 ± 221.3	917.4 ± 37.5

Each value is represented as mean ± S.D. Significance: *p < 0.001* when compared to group A and ^###^ *p < 0.001* when compared to group B.

#### Liver-to-Body Weight Ratio

Table 6 shows the liver-to-body weight ratio in all treatment groups. In the control group, the liver-to-body weight ratio was 3.22, which increased significantly (*p* < 0.001) to 4.81 in the CCl_4_-challenged group, whereas the highest dose of GREE treatment led the ratio to a value of (*p* < 0.001) 3.41.

**TABLE 6:** Effect of GREE on the liver-to-body weight percentage.

Liver wt/bw (%)	Control	GREE 500	CCl_4_	CCl_4_ +GREE 100	CCl_4_+GREE 300	CCl_4_+GREE 500
	3.22 ± 0.4	3.41 ± 0.07^##^	4.81 ± 0.6***	4.01 ± 0.7	3.51 ± 0.45	3.1 ± 0.1

Values are represented as the mean ± S.D., *n* = 4. Significance: ***p < 0.001 when compared to the control group and^##^ *p < 0.001* when compared to the CCl_4_-treated group.

#### Effect of GREE on the mRNA Expression of Genes Related to Liver Fibrosis

**FIGURE 4:**
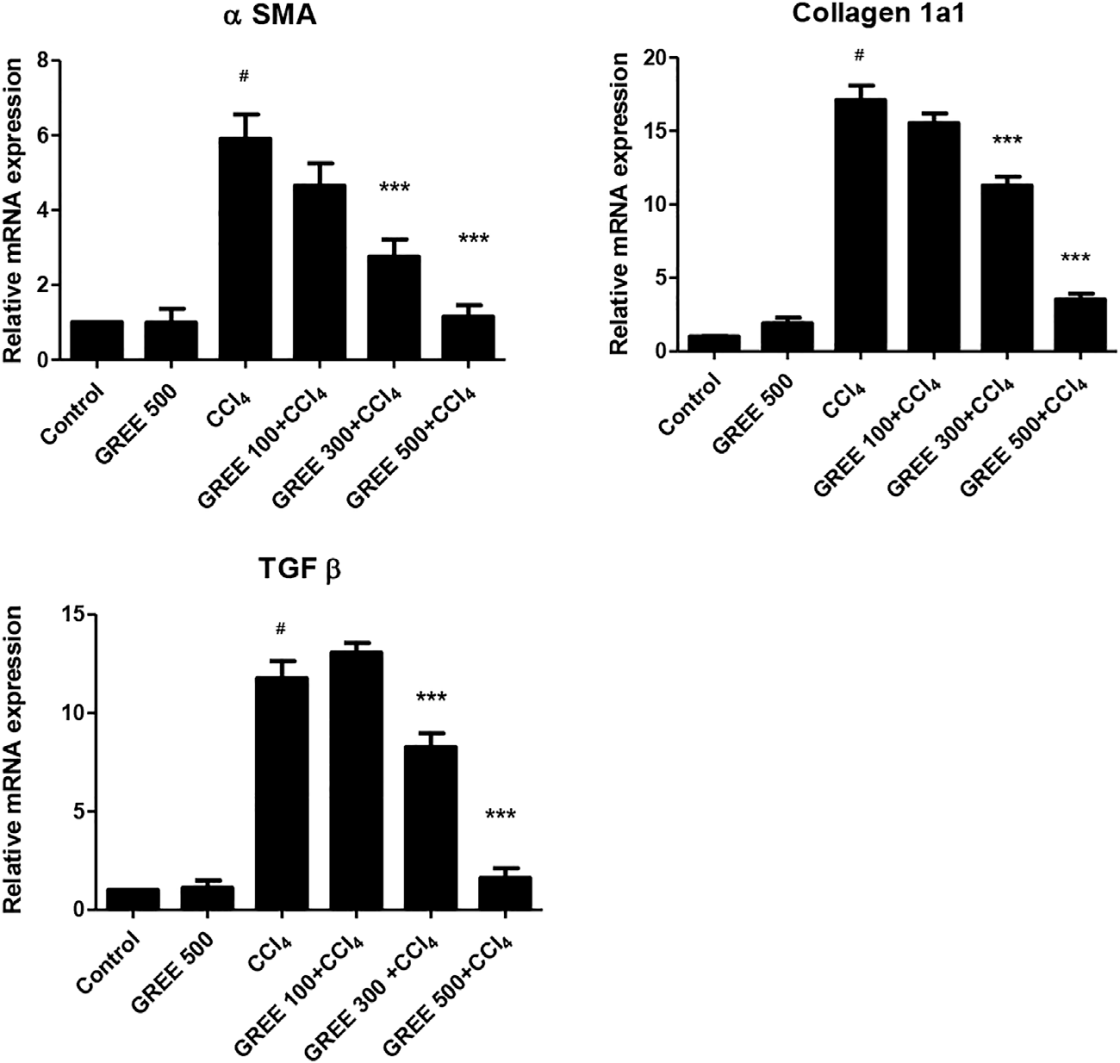
mRNA expression in various treatment groups. The relative mRNA expression of smooth muscle actin, collagen, and TGF-beta in the liver tissues of animal from various groups was determined by real-time PCR. Values are represented as the mean ± S.D., *n* = 4. Significance: ^#^ *p < 0.001* when compared to group A and ***p < 0.001 when compared to group B.

Figure 4 shows the relative mRNA expression of α-SMA in all groups. The mRNA expression of α-SMA in the control group and the GREE only-treated group remained on a similar basal level. However, in CCl_4_, the mRNA expression of α-SMA increased significantly (*p < 0.001*) by sixfold, while in the treatment group, mRNA levels of α-SMA significantly (*p < 0.001*) decreased in a dose-dependent manner. The expressions of Coll in response to CCl_4_ and GREE treatments also showed a similar trend. The mRNA expression of Coll increased by 17-fold in the CCl_4_-challenged group compared with the control group. Similarly, levels of mRNA reduced significantly (*p < 0.001*) in the treatment groups in a dose-dependent manner.

The expressions of TGF-β in response to CCl_4_ and GREE treatments also showed a similar trend. The mRNA expression of Coll increased by 12-fold in the CCl_4_-challenged group compared with the control group. However, the levels of mRNA reduced significantly (*p < 0.001*) only in the treatment groups treated with the higher dose; at the lowest dose (100 mg/kgbw), the impact was not observed.

#### Histology of Liver Tissue

**FIGURE 5:**
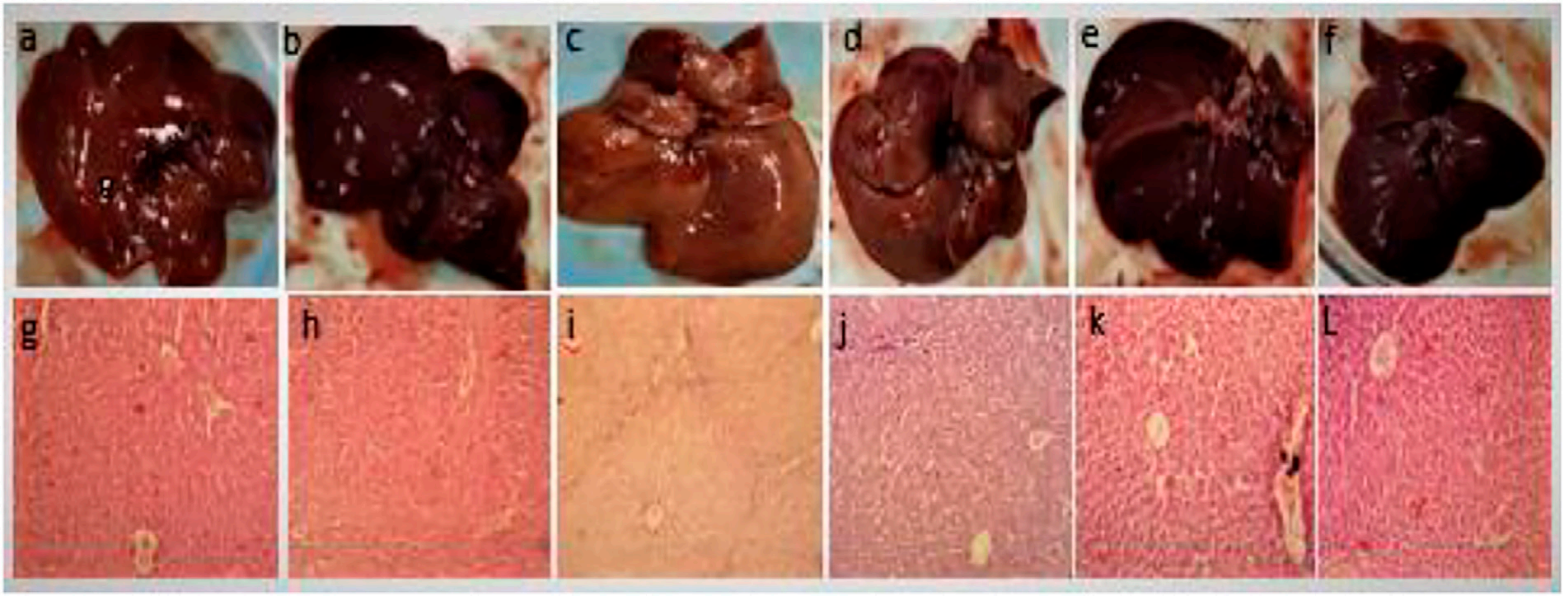
Gross anatomical features of livers. Control group **(A,G)**, GREE-treated group **(B,H)**, CCl_4_-treated group **(C,I)**, CCl_4_+GREE (100 mg/kgbw)-treated group **(D,J)**, CCl_4_+GREE (300 mg/kgbw)-treated group **(E,K),** and CCl_4_+ GREE (500 mg/kgbw)-treated group **(F,L)**. Livers were removed, washed with normal saline, and photographed.

Figure 5 shows the gross structure of liver sections stained with hematoxylin to study microscopic changes in response to CCl_4_ and GREE treatments. Figure 3 shows marked changes in the liver of group B compared to group A. The liver of CCl_4_-treated animals showed coarse and irregular surfaces with nodular texture and appearance, along with a change in color from reddish-brown to light brown. The liver was felt tough by touch, and apparently, the size had shrunken. These features remarkably confirm the onset of cirrhosis in this group. Groups D-F (Figure 2D–F) show the effect of treatment on a gross level. The liver showed comparatively better, smoother surface texture, and regularity in liver lobes was comparable to that of group A.

#### Microscopic Features

Figure 5 lower panel shows H&E-stained liver sections. Figures 5G–H show normal histological architecture of the liver with central vein, healthy hepatocytes, and no immune infiltration. Figure 5H shows necrotic cells with compact central vein, disappearance of nuclei, and portal triad surrounded with marked inter-portal deposition of collagen represented by light blue fibrous tissue that resulted in nodular structure and bridge fibrosis. Figure 5J–L has similar features as that of the control group, and the sign of extracellular matrix deposition was absent.

## Discussions

*G. robusta* has already been reported for containing more than a dozen phytochemicals that have free radical (DPPH) scavenging (or antioxidant) and antiproliferative activities, as shown in cancerous cell lines (Ritchie et al., 1965; Chuang et al., 2011; Wei et al., 2012). The current study shows that GREE has an ability to prevent liver fibrosis and cirrhosis by restricting the induction of molecular markers of fibrosis, such as α-SMA and Coll in CCl_4_-induced liver fibrosis. Earlier studies have shown the presence of epilyoni-resinol, resin glucosides, rhamnocitrin 3-o-rutinoside, 5-n alkyl resorcinol, and macrocyclic phenols in the extracts of G. *robusta*. We detected polyphenols and flavonoids in GREE, so earlier studies are supportive of our findings regarding its active phytochemical constituents as carried out by analysis. Moreover, the GCMS data showed the presence of polyalcohols and unsaturated fatty acids including linoleic acid, which are precursors of many metabolites in plants (Cannon et al., 1970; Cannon et al., 1973; Chuang and Wu, 2007; Chuang et al., 2011).

CCl_4_ induces liver fibrosis *via* activation of the PKC/NF-κB pathway (Toriumi et al., 2013). A recent *in vitro* cell culture study performed by researchers showed that flavonoids possess antifibrotic effects *via* inhibiting the NF-κB pathway of liver inflammation (An et al., 2021). Since the extract of *Grevillea robusta* contained high content of flavonoids (about 15%), we presume that the antifibrotic effects observed in our experiments are due to the flavonoids.

The CCl_4_-induced model of liver damage and fibrosis has been studied many times to explore plant-based hepatoprotective drugs (Atta *et al.*). CCl_4_ induces liver damage by generating CCl₃^°^ radical that interferes with proteins and lipids and consequently damages the hepatocytes (Chhimwal et al., 2020). During a short-term exposure, CCl_4_ results in liver steatosis by inhibiting the secretion of triacylglycerol, while its long-term exposure leads to the initiation of oxidative stress–induced inflammatory processes and hepatocyte damage (Boll et al., 2001). Hence, CCl_4_ leads to liver steatosis followed by fibrosis, which is characterized by the deposition of ECM and a concomitant release of growth factors and cytokines. Hepatic stellate cells (HSCs) contributed majorly to liver fibrosis because they promote the deposition of collagen on the ECM and produce other markers such as α-SMA (alpha-smooth muscle actin) and collagen alpha 1 (Liu et al., 2019). These markers play a vital role in liver fibrosis generation. The therapeutic strategy that inhibits HSC activation is becoming a promising therapy for the treatment of liver fibrosis (Martin-Mateos et al., 2019). Transforming growth factor-beta (TGF-β) is a fibrogenic cytokine activated by the HSC activation and binds to their receptors on cell surface and starts the activation of other transcription factors such as Smad2, collagen alpha 1, and myofibroblasts, thereby promoting hepatic fibrosis (Liu et al., 2019).

Hepatocyte growth factor and TGF-β primarily have become evident in triggering the hepatocyte growth and regeneration due to oxidative stress (Tiggelman et al., 1995; George et al., 1999; Friedman, 2003; Canbay et al., 2004; Inagaki et al., 2005). In our study, the results showed that GREE highly significantly decreased the mRNA expression analysis when compared to the disease group. A significant reduction in the mRNA expression of the main three markers (TGF-β, α-SMA, and Collα1) supported our hypothesis that GREE may play an important role in the hepatic protection from the liver fibrosis.

ALT and AST are intracellular enzymes and concentrated in many parts of the body, including the liver, kidneys, brain, and skeletal, cardiac, and muscle cells, while GGT is present in the cholangiocytes of the bile duct in the liver. The serum levels of ALT, AST, and GGT are increased during liver disease (Kew, 2000). The data obtained in this study align with the earlier observations as CCl_4_ administration induced necrosis of hepatocytes with the release of AST, ALT, and GGT in the serum of CCl_4_-challenged animals (Nwaehujor and Udeh, 2011; Hussain et al., 2017; Rehman et al., 2017).

Hematological abnormalities have also been demonstrated during liver damage, fibrosis, and cirrhosis. An increase in the WBC count clearly shows an onset of inflammation. Increase in WBC during liver inflammation has already been demonstrated previously (Saba et al., 2010). GREE treatment in CCl_4_-challenged rats has shown an inclination toward normalization with respect to WBC and RBC counts though it did not reach a statistical significance level. Restoring the RBC count might be due to an increased erythropoietin expression during fibrosis reversal as suggested by others (Yang et al., 2003).

The chemical composition and amount of ECM undergo profound variations and reorganization during liver fibrosis and its resolution (Tsukada et al., 2006). During liver injury, the fibrotic liver can contain almost six times more ECM than a normal healthy liver. ECM is chiefly produced by HSC, which becomes highly proliferative and produces large masses of Coll (Tiggelman et al., 1995; Lindquist et al., 2000), and parallel to this, enzyme-regulated degradation of ECM is suppressed (Arthur, 2000). Additionally, activated HSCs are characterized by increased levels of cytoskeleton proteins such as α-SMA (Bataller and Brenner, 2001; Xu et al., 2014).

The RT-PCR data obtained in this study support earlier findings that CCl_4_ caused a significant increase in the expression of α-SMA and Coll mRNA (Atta et al.). Treatment with GREE lowered the mRNA expression levels of α-SMA and Coll. During liver fibrosis, the mRNA expression regulation in HSCs is a dynamic process, which increases to a peak and then either stabilizes or decreases. Thus, the mRNA expression response mainly depends on factors such as the severity of disease and the time lapse. It has already been documented (Boll et al., 2001) that steatosis is an earlier phase of liver damage, followed by fibrosis after CCl_4_ administration. In the treatment groups, the process of liver damage was restricted as compared to the CCl_4_-challenged group, which is supported by H&E staining (Figure 5). GREE-treated animals also showed a marked reduction in ECM deposition around the portal area, which suggests GREE treatment slowed down the damaging effect of CCL_4_.

Gross anatomical observations (Figure 5 top panel) and the liver-to-body mass ratio (Table 6) also support the PCR data, which show that GREE has an ameliorative effect on fibrotic tissue accumulation in the liver of CCL_4_-challenged rats. The absence of nodular structure in the livers of animals from treatment groups confirms the prevention of cirrhosis. Similarly, the restoration of the liver-to-body mass ratio indicates the preventive role of GREE in CCl_4_-induced liver fibrosis and cirrhosis.

Oxidative stress has an important role in the hepatocyte damage, activation of HSC, and induction of fibrosis. Flavonoids and polyphenols have been reported for their antioxidant properties. Our study also demonstrated the presence of flavonoids, polyphenols, and antioxidant activity in GREE. Therefore, hepatoprotective and fibrosis delaying effects can be attributed to the antioxidant potential of polyphenols and flavonoids present in the extract (GREE).

Cumulatively, phytochemical, biochemical, molecular, and histopathological data suggest a hepatoprotective and an antifibrotic role of GREE in prophylaxis and the treatment of CCL_4_-intoxicated liver of the rats.

### Future Prospects

This study is unique in a way that *G. robusta* extract effects have been demonstrated for the first time on molecular levels of α-SMA and Coll in addition to *in vitro* antioxidant activity. In the future, we are planning to perform bioassay-guided isolation and characterization of the antifibrotic constituents from *G. robusta* using the *in vitro* cell culture of primary hepatic stellate cells or myofibroblasts. We also intend to compare the mRNA expression levels with protein levels of α-SMS, Coll, and TGF-β and some other markers using immunofluorescence and Western blotting techniques.

## Data Availability

The raw data supporting the conclusions of this article will be made available by the authors, without undue reservation.
